# The Chemistry and Biochemistry of Myochrisine

**DOI:** 10.1155/MBD.1994.529

**Published:** 1994

**Authors:** Colin J. L. Lock, Helen E. Howard-Lock, Walter F. Kean, Daren J. LeBlanc, Zhixian Wang

**Affiliations:** 1 Laboratories for Inorganic Medicine Departments of Chemistry, Medicine and Pathology McMaster University, ABB-266A Hamiton ON L8S 4M1 Canada


					THE CHEMISTRY AND BIOCHEMISTRY OF MYOCHRISINE
Colin J. L.Lock, Helen E. Howard-Lock, Walter F. Kean,
Daren J. LeBlanc and Zhixian Wang
Laboratories for Inorganic Medicine, Departments of Chemistry, Medicine and Pathology,
McMaster University, ABB-266A, Hamiton, ON L8S 4M1, Canada
As part of our studies of the interaction of gold(I) with thiols and other sulphur-containing molecules
we have managed to isolate and characterize by X-my crystallography, the first gold(I)-thiomalate
complex. We shall describe this work and speculate on its significance to the nature of the gold
drug myochrysine, and its mode of biochemical action.
529

				

